# Identification of Proteins of Tobacco Mosaic Virus by Using a Method of Feature Extraction

**DOI:** 10.3389/fgene.2020.569100

**Published:** 2020-10-09

**Authors:** Yu-Miao Chen, Xin-Ping Zu, Dan Li

**Affiliations:** Information and Computer Engineering College, Northeast Forestry University, Harbin, China

**Keywords:** feature extraction, physicochemical properties, identification, tobacco mosaic virus, machine learning

## Abstract

Tobacco mosaic virus, TMV for short, is widely distributed in the global tobacco industry and has a significant impact on tobacco production. It can reduce the amount of tobacco grown by 50–70%. In this research of study, we aimed to identify tobacco mosaic virus proteins and healthy tobacco leaf proteins by using machine learning approaches. The experiment's results showed that the support vector machine algorithm achieved high accuracy in different feature extraction methods. And 188-dimensions feature extraction method improved the classification accuracy. In that the support vector machine algorithm and 188-dimensions feature extraction method were finally selected as the final experimental methods. In the 10-fold cross-validation processes, the SVM combined with 188-dimensions achieved 93.5% accuracy on the training set and 92.7% accuracy on the independent validation set. Besides, the evaluation index of the results of experiments indicate that the method developed by us is valid and robust.

## Introduction

Tobacco mosaic virus is worldwide distribution and is the furthest invasive virus which is most harmful to crops. Tobacco is one of the important economic crops in our country, however, the existence of tobacco mosaic disease has greatly reduced the yield and quality of tobacco. Since plants do not have a complete immune system, once infected, the leaves can show mosaic symptoms or even deformities and the growth can also be chronically diseased, which makes tobacco mosaic virus is very difficult to control (Hu and Lee, 2015).

The study of viruses has attracted many scholars, and with the development of computer machine learning algorithms, many scholars have applied machine learning algorithms to the study of viruses. Metzler and Kalinina (2014) used one-class SVM method to detect atypical genes in viral families based on their statistical features, without the need for explicit knowledge of the source species. The simplicity of the statistical features used allows the method to be applied to a variety of viruses. Salama et al. (2016) predicted new drug-resistant strains that facilitate the design of antiviral therapies. In this study, neural network techniques were used to predict new strains, and using a rough set theory based on algorithm to extract these points mutation patterns. For phage virion proteins (PVPs) prior to *in vitro*, Manavalan et al. (2018) developed a SVM-based predictor that exhibited good performance and avoided the expensive costs required for experiments.

Using biochemical experiments to study all tobacco mosaic virus is a challenge because it is expensive and a waste of researchers' time, and there is no specific predictor to predict tobacco mosaic virus. So, in this research we evaluated the predictive performance of different classifiers in combination with different feature extraction methods. We have chosen classical machine learning algorithms and classical feature extraction methods. The feature extraction methods AAC (Chou, 2001), 188-dimensions (Dubchak et al., 1995) and CKSAAGP (Chen et al., 2018) and their combination were chosen for the reasons. AAC is the first proposed feature extraction method that is widely used to predict the function of proteins. It based on their amino acid composition. CKSAAGP describes the spatial distribution information of amino acids, 188 dimensions in addition to the physicochemical properties of amino acids. Three feature extraction methods from different aspects, so these three feature extraction methods were chosen. The combined feature extraction method was attempted considering the expectation of better results. We finally chose the combination of support vector machine (SVM) with 188-dimensions as the final predictor because it has the best prediction effect.

## Materials and Methods

**Figure 1:**
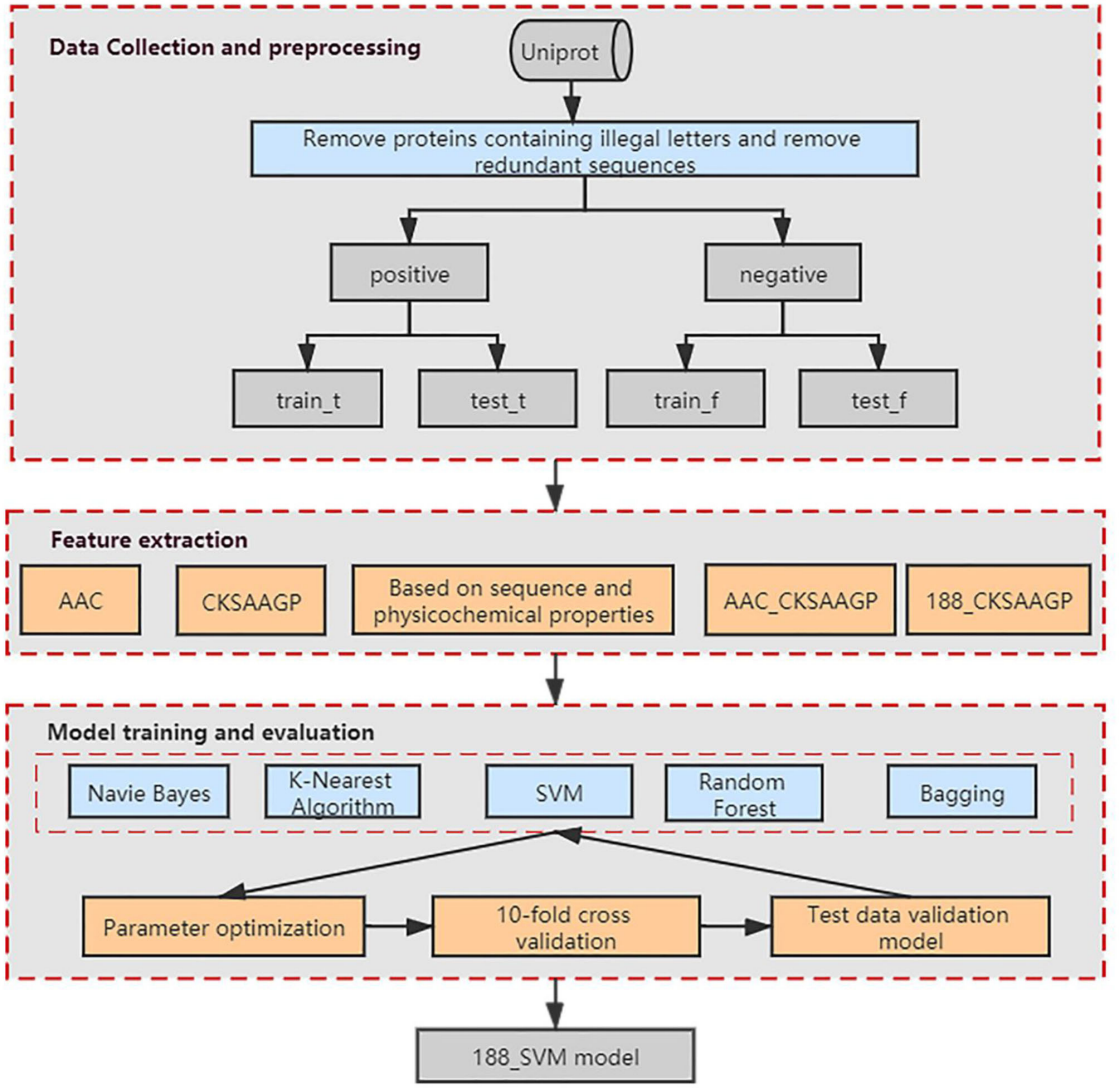
Schematic showing the workflow used to develop our method. AAC, CKSAAGP, 188-dimensions, AAC_CKSAAGP, 188_ CKSAAGP are our five feature extraction methods. Naive Bayes, K-Nearest Algorithm, SVM, Random Forest and Bagging are our five classification algorithms.

Our method was developed based on three steps (Figure 1). Step 1: we collected the data and preprocessed the dataset to obtain a non-redundant benchmark dataset that does not contain non-standard characters. Step 2: We used Amino Acid Composition (AAC), feature extraction method based on the composition of amino acid sequence and physicochemical properties (188-dimensions), composition of k-spaced amino acid pairs (CKSAAGP), and the combined methods AAC_CKSAAGP and 188_CKSAAGP which are proposed in this paper to extract features from protein sequences. Step 3: Five algorithms and 10-fold cross-validation are used to build and estimate the models, which are Random Forest (RF), Bagging, K-Nearest Neighbor (KNN), Naive Bayes (NB), and Support Vector Machine (SVM). Then, we validated experimental results using an independent validation set.

## Benchmark Dataset

High-quality baseline data sets contribute to the accuracy of model predictions (Yang et al., 2019b; Cheng et al., 2020; Zhu et al., 2020). The dataset obtained for this experiment was derived from the Swiss-Prot database in The Uniprot (2018). Firstly, we used the keyword search method to collect data from the UniProt database. By entering the keywords “Tobacco mosaic virus” and “Tobacco leaf not virus” to obtain the positive and negative data needed for the experiment. For the sake of improving the reliability of the data, the following operations were performed: (1), Deleted the protein sequences containing non-standard letters, i.e. “B,” “X,” “Z,” etc.; thus, we obtained 5,309 protein sequences of tobacco mosaic virus and 45,827 protein sequences of non-tobacco mosaic virus. (2), If the sample contains multiple similarity sequences, this sample is not statistically representative. We used the CD-HIT program (Fu et al., 2012) to delete sequences with similarity surpass 40% in positive and negative data sets (Zou et al., 2018). After removing the redundant sequences, we eventually obtained a dataset of 715 protein sequences of TMV proteins and 17,983 protein sequences of tobacco leaf proteins.

There are 715 sequences in the positive datasets and 17,893 sequences in the negative datasets. Much more negative data than positive data. For the purpose of balancing the datasets, we took a downsampling approach. We split the negative data by the size of the positive data. And randomly selected 10 of these copies as the negative dataset, so we obtained a negative dataset containing 7,150 sequences. The resulting positive and negative datasets were divided proportionally. The final training dataset consists of 500 positive data and 5,000 negative data. The test dataset consists of 215 positive data and 2,150 negative data. These data are available in our software package.

## Feature Extraction

Feature selection will affect the performance of machine learning methods for bioinformatics problems (Zhao et al., 2015). In the research of this paper, five feature extraction methods are selected, including amino acid composition (AAC), composition of k-spaced amino acid pairs (CKSAAGP), 188-dimensions feature extraction method, and the combined methods AAC_CKSAAGP and 188_CKSAAGP which are proposed in this paper.

### Amino Acid Composition (AAC)

The coded amino acid composition coding scheme (Bhasin and Raghava, 2004) calculates the probability of occurrence of 20 natural amino acids (i.e., “ACDEFGHIKLMNPQRSTVWY”) in protein sequences or peptide chains (Zhong et al., 2020). The calculation formula for each amino acid is as follows:

(1) $$\begin{array}{r} v_{i}=\frac{c_{i}}{len\left(seq\right)},i\in \left(A,C,\cdots \;,Y\right) \end{array}$$

Where *c*_*i*_ and *len*(*seq*) represent the number of occurrences of amino acid i in the sequence or peptide chain and the length of the sequence or peptide chain, respectively (Lin et al., 2005; Lv et al., 2020).

### Composition of k-Spaced Amino Acid Pairs (CKSAAGP)

The composition of K-spaced amino acid pairs (Chen et al., 2018) can be regarded as a variant of CKSAAP, which calculates the frequency of amino acid pairs separated by any k residues (the default maximum for k is set to 5) (Chen et al., 2020). Taking K = 0 as an e.g., a feature vector is defined as:

(2) $$\begin{array}{r} \gamma_{0}={\left(\frac{C_{g1g1}}{C_{n}},\frac{C_{g1g2}}{C_{n}},\frac{C_{g1g3}}{C_{n}},\cdots \;,\frac{C_{g5g5}}{C_{n}}\right)}_{25} \end{array}$$

wherein, (*g*1*g*1, *g*1*g*2, *g*1*g*3, ⋯*g*5*g*5) represents 0-spacing amino acid group pairs. There are 25 groups in total, and each descriptor represents the composition of the corresponding residue pair in the protein sequence (Zhu et al., 2020). *C*_*g*1*g*1_ (Zhang et al., 2014) represents the number of times the residue pair *g*1*g*1 appears in the sequence and Cn represents the total number of residue pairs with a gap of 0 in the sequence. In a protein sequence of length N, for different values of K, the value of n can be defined as:

(3) $$\begin{array}{r} n=P-K-1,K\in \left(0,1,2,3,4,5\right) \end{array}$$

When K takes each value, the CKSAAGP feature vector (γ_0_, γ_1_, γ_2_, γ_3_, γ_4_, γ_5_) has a total size of 150 dimensions.

### 188-Dimensions

Each amino acid sequence has different physical and chemical properties including amino acid composition, hydrophobicity, normalized Van der Waals volume, polarity, polarizability, charge, surface tension, secondary structure, and solvent accessibility for each residue in the sequence (Cai et al., 2003). The feature extraction method for protein P is formulated as follows:

(4) $$\begin{array}{r} P=\left\{C,Tr,D\right\} \end{array}$$

Where C represents the frequency of a kind of specific attribute (such as polarity) amino acid appearing in the global sequence. Tr represents the global percentage of transitions between a specific amino acid and another amino acid of a specific property. D is used to describe the first, 25%, 50%, 75% and last position of each specific amino acid in the peptide chain.

Assume that the protein sequence “VNVVVNVNNVVVVVNVNNNVVNNVNNNVVNVN” has 17 valines (*n*_1_ = 17) and 15 asparagines (*n*_2_= 15). The components of these two amino acids are $\frac{n_{1}}{n_{1}+n_{2}}\times 100.00=53.13$ and $\frac{n_{2}}{n_{1}+n_{2}}\times 100.00=46.87,$ respectively. The number of transitions from V to N or N to V is 17, so the percentage of these transitions is (17/32) × 100.00 = 53.13. The first, 25, 50, 75% and last positions of valine in the peptide chain are 1, 5, 13, 21, 31, respectively, so the D-attribute of valine is *D*_*V*_ = (3.13, 15.63, 40.63, 65.23, 96.88). In the same way, the position of asparagine in the peptide chain can be found. In summary, the amino acid composition descriptors are C = (53.13, 64.87), Tr = (53.13), and D = (3.13, 15.63, 40.63, 65.23, 96.88, 6.25, 28.13, 68.75, 78.13, 100.00). Descriptors of other attributes can be described through a similar process, and then all the descriptors are combined to form a 188-dimensions feature vector.

**Table:** 

Sequence	V	N	V	V	V	N	V	N	N	V	V	V	V	V	N	V	N	N	N	V	V	N	N	V	N	N	N	V	V	N	V	N
Sequence index	1				5					10					15					20					25					30		
Index for V	1		2	3	4		5			6	7	8	9	10		11				12	13			14				15	16		17	
Index for N		1				2		3	4						5		6	7	8			9	10		11	12	13			14		15
V/N transitions	|	|			|	|	|		|					|	|	|			|		|		|	|			|		|	|	|	

### Combined Method

A combination of AAC, CKSAAGP, and feature extraction based on a combination of sequence and physicochemical properties constitute a new feature extraction method. The number of characterization dimensions for the AAC_CKSAAGP combination is 170, and the number of characterization dimensions for the 188_CKSAAGP combination is 338. Since the information of amino acid content is included in the 188-dimensions feature extraction method, the combination of AAC and 188-dimensions feature extraction method is not used in this paper.

### Classifier

In this paper, five classifiers were used for the experiments. these classifiers were implemented through Waikato Environment for Knowledge Analysis software (Azuaje et al., 2006).

### Random Forest

Random Forest (RF) is an integrated learning method first proposed by Leo Breiman and Adele Cutler (Azuaje et al., 2006; Goldstein et al., 2011; Cheng et al., 2018b,d), and it is a combination of multiple decision trees. Nowadays, many bioinformatics' problems use Random Forest (Tastan et al., 2012; Jamshid et al., 2018; Lyu et al., 2019; Ru et al., 2019; Lv et al., 2020). For processing large amounts of data, Random Forest is characterized by high accuracy, high speed and good robustness. In RF, we need to input the prediction samples into each tree for prediction, and finally use the voting algorithm to determine the result of prediction. The voting algorithm is shown speed and good robustness. The anti-noise capability of RF is strong, and it often shows good robustness when processing high-dimensional data. The voting algorithm is shown below:

(5) $$\begin{array}{r} \text{result}=\text{sgn}\left(\overset{\text{n}}{\underset{\text{i}=1}{\sum }}\text{pr}{\text{e}}_{\text{label}}\right) \end{array}$$

Where result is the final prediction, *pre*_*label*_ represents the predicted result for each decision tree, which equals to 1 or −1, and n represents the number of Decision Trees in the model.

### Support Vector Machine

Support Vector Machine (SVM) is often applied to classification problems and is a supervised learning approach (Huang et al., 2012; Jiang et al., 2013; Xing et al., 2014; Kumar et al., 2015; Zhao et al., 2015; Liao et al., 2018; Wang et al., 2019). There are already a number of software packages that support the SVM algorithms. In this experiment, we used Libsvm (Chang and Lin, 2007) in Weka (version 3-8-2) (Hall et al., 2008) to implement the SVM, where we chose RBF, a radial basis function, to classify the proteins of tobacco mosaic virus. Then we determined the regularization parameter C and the kernel parameter g through grid search and 10-fold cross-validation (Wang et al., 2011).

### K-Nearest Neighbor

The K-Nearest Neighbor (KNN) algorithm (Zhang and Zhou, 2007; Lan et al., 2013; Deng et al., 2016) which is one of the simplest, most convenient, and highly effective algorithms. Now it has been frequently used in the functional classification of proteins problems. The key step of KNN prediction is to find the K neighbors closest to the test data from the training set, and then use the category with the most K neighbors as the final category of the test data. In this experiment, we adopt the KNN algorithm based on the Harmanton distance and the Harmanton distance formula is summarized as follows:

(6) $$\begin{array}{r} L_{1\left(x_{i},x_{j}\right)=}\overset{n}{\underset{k=1}{\sum }}|x_{i}^{\left(k\right)}-x_{j}^{\left(k\right)}| \end{array}$$

where: $x_{i}^{k}$(k=1, 2, 3, ……n) is characteristic of the training set and $x_{j}^{k}$(k=1, 2, 3, ……n) is characteristic of the test dataset.

### Naive Bayes

The Naive Bayes (NB) is a easily understand classification algorithm (Xue et al., 2006; Wang et al., 2008; Feng et al., 2013), which is based on the Bayesian classifier and assumes that the feature attributes of the data are simple and independent. In the classification scenario, it greatly reduces the complexity of the Bayesian classification algorithm. Suppose the sample data set is:

(7) $$\begin{array}{r} \left(x_{1}^{\left(1\right)},x_{2}^{\left(1\right)},\cdots \;,x_{n}^{\left(1\right)},s_{1}\right),\left(x_{1}^{\left(2\right)},x_{2}^{\left(2\right)},\cdots \;,x_{n}^{\left(2\right)},s_{2}\right),\\ \cdots \left(x_{1}^{\left(m\right)},x_{2}^{\left(m\right)},\cdots \;,x_{n}^{\left(m\right)},s_{m}\right) \end{array}$$

there are m samples and each sample have n features. The data set has a total of class variable. Generally speaking, the m samples can be classified into s categories, where n features are independent of each other. The category of S is defined as follows:

(8) $$\begin{array}{r} S=\left\{s_{1},s_{2},s_{3},\cdots \;,s_{m}\right\} \end{array}$$

Among them, there are M class variables in the set S. Naive Bayes formula is defined as follows:

(9) $$\begin{array}{r} P\left(y_{i}\text{|}x_{1},x_{2},\cdots \;,x_{n}\right)=\frac{P_{y_{i}}\prod_{j=1}^{n}P\left(x_{j}\text{|}s_{i}\right)}{\prod_{j=1}^{n}P\left(x_{j}\right)} \end{array}$$

### Bagging

Bagging is a typical integrated learning algorithm (Abellán et al., 2017), which is directly based on autonomous sampling. For the input sample set *D* = {(*x*_1_, *y*_1_), (*x*_2_, *y*_2_), ⋯ , (*x*_*m*_, *y*_*m*_)}, a weak learner algorithm is used to classify each time, and a total of T iterations are made, and finally we will obtain a powerful classifier. Since it samples each training model, it has a strong generalization ability that can significantly reduce the variance of the training model.

## Performance Evaluation

There are five main parameters (Kou and Feng, 2015) to evaluate the predictive performance of this experiment, namely, sensitivity (Sn), specificity (Sp) (Ding et al., 2012; Tan et al., 2019), accuracy (ACC) (Thakur et al., 2016; Cheng et al., 2018a,b), Matthews correlation coefficient (MCC) (Yang et al., 2019a,b) and area of ROC curve (AUC) (Lobo et al., 2008; Li and Fine, 2010; Wang et al., 2010; Hajian-Tilaki, 2014; Baratloo et al., 2015). Defined as follows:

(10) $$\begin{array}{rcl} S_{n} & = & \frac{TP}{TP+FN} \end{array}$$

(11) $$\begin{array}{r} S_{P}=\frac{TN}{TN+FP} \end{array}$$

(12) $$\begin{array}{r} ACC=\frac{TP+TN}{TP+TN+FP+FN} \end{array}$$

(13) $$\begin{array}{r} MCC=\frac{\left(TP+TN\right)-\left(FP+FN\right)}{\sqrt{\left(TP+FP\right)\times \left(TN+FN\right)\times \left(TP+FN\right)\times \left(TN+FP\right)}} \end{array}$$

(14) $$\begin{array}{r} AUC=\frac{\frac{\sum_{i\in positiveClAss}rank_{i}-M\times \left(M+1\right)}{2}}{M\times N} \end{array}$$

Where TP represents the amount of tobacco mosaic virus correctly predicted by the model (Dong et al., 2015); TN indicates the amount of non-tobacco mosaic virus correctly predicted by the model (Niu et al., 2018); FN indicates the amount of non-tobacco mosaic virus incorrectly predicted by the model (Kim et al., 2016); FP indicates the amount of non-tobacco mosaic virus predicted by the model; M and N indicate the amount of positive and negative data, respectively; and *rank*_*i*_ is the score of the i-th positive sample was calculated by classification. The higher the value of the five evaluation indicators above, the better the model prediction.

## Results and Discussion

### Performance Evaluation of Different Classifiers

**Figure 2:**
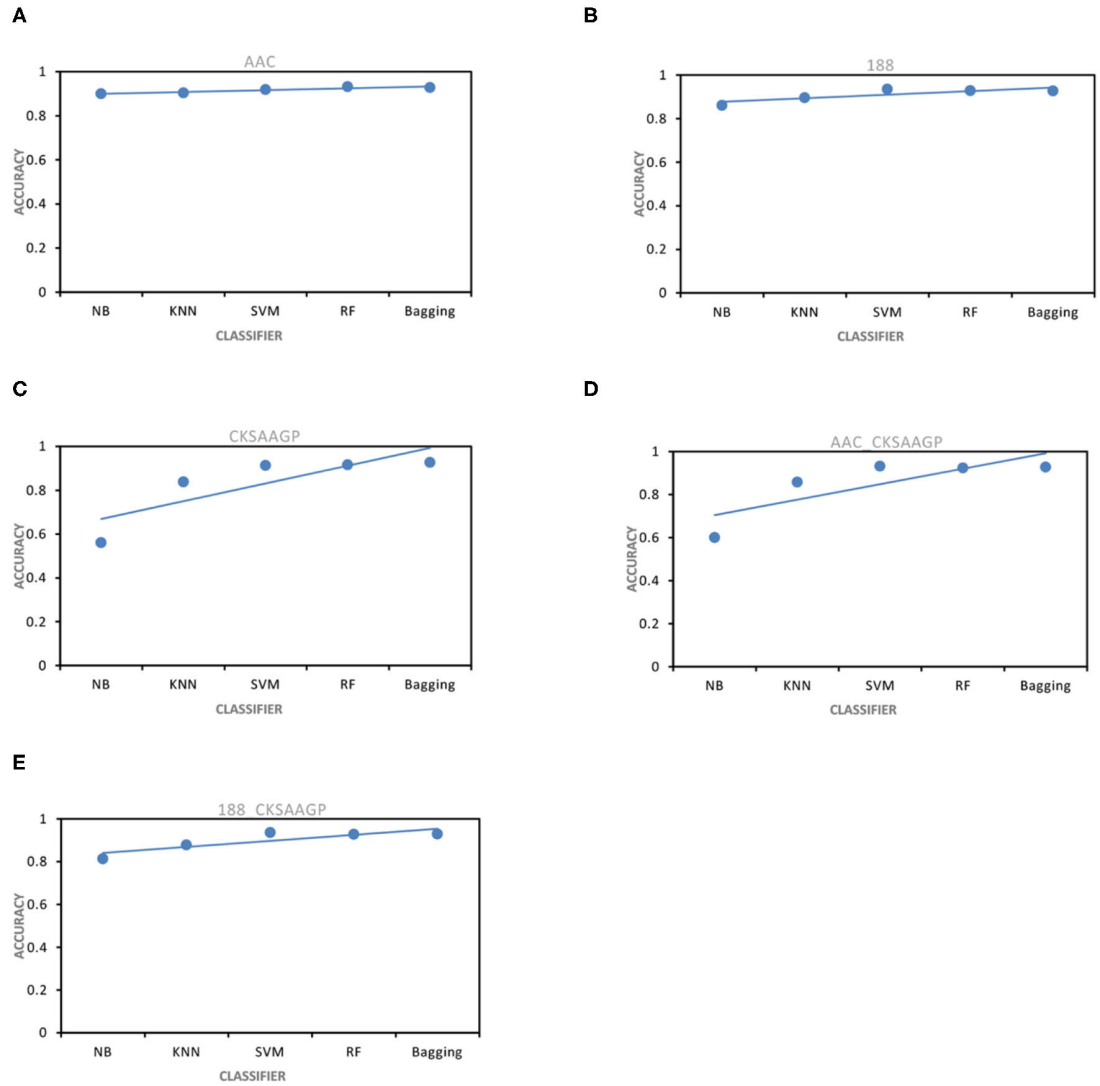
ACC obtained by five feature extraction methods [**(A–E)** AAC, 188-dimensions, CKSAAGP, AAC_CKSAAGP, and 188-CKSAAGP] combined with five classifiers (NB, KNN, SVM, RF, Bagging) through 10-fold cross-validation.

**Figure 3:**
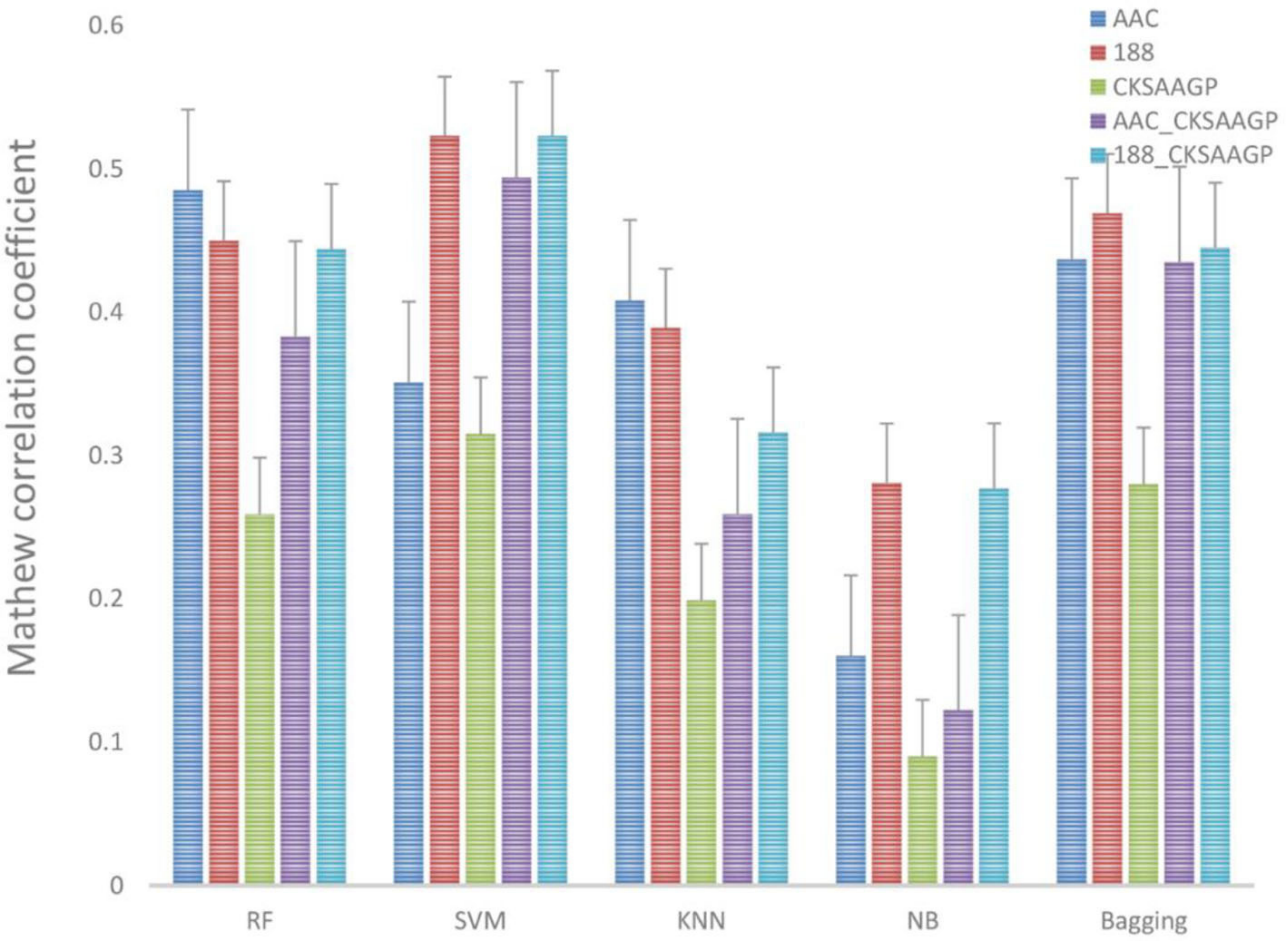
Different feature extraction methods were used to predict the performance of different classification methods. The average MCC value of the classifier was tested by 10-fold cross-validation.

The ACC and MCC of SVM and RF were mostly higher than the predictors of NB, KNN and Bagging under different feature extraction methods (Figures 2, 3). When the feature extraction method selects 188_CKSAAGP or 188-dimensions, SVM reach the highest ACC. When the feature extraction method uses AAC, RF achieves the highest ACC. Through 10-fold cross-validation, the MCC of SVM is higher than that of RF (Figure 3).

However, when comparing predictor superiority, it is possible to use not only the predicted ACC and MCC comparison, but also the trade-off between Sn and Sp. The sensitivity (Sn) and specificity (Sp) of SVM, RF, and Bagging predictor variables are greater than those of NB and KNN (Table 1 and **Figure 5**). This result shows that SVM, RF, and Bagging predict tobacco mosaic virus are better than NB and KNN due to the difference in the ability of these five common classification algorithms to handle multidimensional datasets. NB is a naive algorithm based on the assumption that the individual properties are independent of each other, and NB is very friendly to low dimensional features. However, for multidimensional datasets, there is often some correlation between attribute features. The low ACC of KNN may be because the small size of the training datasets. The SVM, RF and Bagging classification algorithms do not require much in terms of dataset dimensionality, and they can handle high-dimensional, noisy and missing datasets with strong correlation between attributes.

**Table 1:** Predictive effect of NB, KNN, SVM, RF, and Bagging on different trait extraction methods for tobacco mosaic virus.

**Method**	**Feature**	**Sp**	**Sn**	**ACC (%)**	**MCC**	**AUC**
NB	AAC	0.866	0.899	89.95	0.16	0.719
KNN		0.902	0.905	90.45	0.408	0.699
SVM (c = 9, g = 3)		0.901	0.918	91.80	0.351	0.61
RF		**0.932**	**0.932**	**93.22**	**0.485**	**0.833**
Bagging		0.922	0.928	92.76	0.437	0.799
NB	188-dimensions	0.882	0.862	86.16	0.281	0.732
KNN		0.899	0.897	89.65	0.389	0.699
SVM		**0.936**	**0.936**	**93.58**	**0.523**	0.657
RF		0.929	0.929	92.91	0.45	**0.838**
Bagging		0.924	0.93	93.04	0.469	0.807
NB	CKSAAGP	0.859	0.561	56.05	0.09	0.64
KNN		0.868	0.839	83.87	0.199	0.618
SVM (c = 5, g = 3)		0.894	0.914	91.38	**0.315**	0.601
RF		0.917	0.916	91.58	0.259	**0.779**
Bagging		**0.919**	**0.917**	**91.69**	0.28	0.711
NB	AAC_CKSAAGP	0.866	0.601	60.07	0.122	0.669
KNN		0.878	0.858	85.82	0.259	0.648
SVM (c = 3, g = 3)		0.923	**0.931**	**93.15**	**0.494**	0.675
RF		**0.927**	0.924	92.36	0.383	**0.831**
Bagging		0.921	0.927	92.75	0.435	0.795
NB	188_ CKSAAGP	0.885	0.814	81.40	0.277	0.726
KNN		0.887	0.878	87.80	0.316	0.669
SVM (c = 3, g = −11)		**0.936**	**0.936**	**93.58**	**0.523**	0.657
RF		0.929	0.929	92.85	0.444	**0.823**
Bagging		0.925	0.93	93.00	0.463	0.803

*Sp, specificity; Sn, sensitivity; ACC, accuracy; MCC, Matthews correlation coefficient; AUC, area of ROC curve. AAC, amino acid composition; 188-dimensions, composition based on sequence and physicochemical properties; CKSAAGP, composition of k-spaced amino acid pairs; AAC_CKSAAGP, combination of AAC and CKSAAGP; 188_CKSAAGP, combination of 188-dimensions and CKSAAGP. The bold values represent the highest score of current feature extraction method in different classifiers*.

In addition, we also used the test datasets to verify the model. The results are shown in Table 2. The results show that the model constructed by SVM combined with 188-dimensions or 188_CKSAAGP achieves a high AAC, which shows that this model is reliable. Although the model constructed by SVM combined with 188-dimensions or 188_CKSAAGP is lower than other algorithms in terms of AUC, evaluation indicators such as Sp, Sn, and MCC have all achieved the best results. Therefore, the SVM algorithm is very promising in TMV classification. Due to the above reasons, this experiment chose SVM as the final classifier to predict TMV.

**Table 2:** Through the use of test data, the evaluation results of the prediction model of NB, KNN, SVM, RF, and Bagging combined with different types of extraction methods.

**Method**	**Feature**	**Sp**	**Sn**	**ACC(%)**	**MCC**	**AUC**
NB	AAC	0.845	0.89	0.8905	0.051	0.677
KNN		0.896	0.901	0.9011	0.368	0.673
SVM		0.893	0.914	0.9142	0.289	0.508
RF		**0.912**	**0.923**	**0.9226**	**0.372**	**0.781**
Bagging		**0.912**	0.922	0.9222	**0.372**	0.734
NB	188-dimensions	0.877	0.859	0.8588	0.255	0.709
KNN		0.886	0.888	0.8875	0.311	0.653
SVM		**0.925**	**0.928**	**0.9277**	**0.434**	0.615
RF		0.92	0.924	0.9243	0.393	**0.799**
Bagging		0.909	0.922	0.9218	0.377	0.774
NB	CKSAAGP	0.858	0.558	0.5577	0.086	0.634
KNN		0.86	0.832	0.8321	0.15	0.587
SVM		0.886	0.91	0.9099	**0.268**	0.582
RF		**0.922**	**0.915**	0.9146	0.235	**0.736**
Bagging		0.917	**0.915**	**0.9150**	0.243	0.692
NB	AAC_CKSAAGP	0.861	0.589	0.5886	0.104	0.647
KNN		0.87	0.859	0.8592	0.213	0.615
SVM		0.915	**0.926**	**0.9256**	**0.423**	0.63
RF		**0.922**	0.919	0.9188	0.312	**0.777**
Bagging		0.915	0.923	0.9226	0.373	0.726
NB	188_CKSAAGP	0.879	0.811	0.8106	0.244	0.705
KNN		0.877	0.87	0.8702	0.257	0.636
SVM		**0.925**	**0.928**	**0.9277**	**0.434**	0.615
RF		0.92	0.925	0.9247	0.399	**0.776**
Bagging		0.915	0.924	0.9239	0.393	0.75

*The bold values represent the highest score of current feature extraction method in different classifiers*.

### Performance Evaluation of Different Feature Extraction Methods

**Figure 4:**
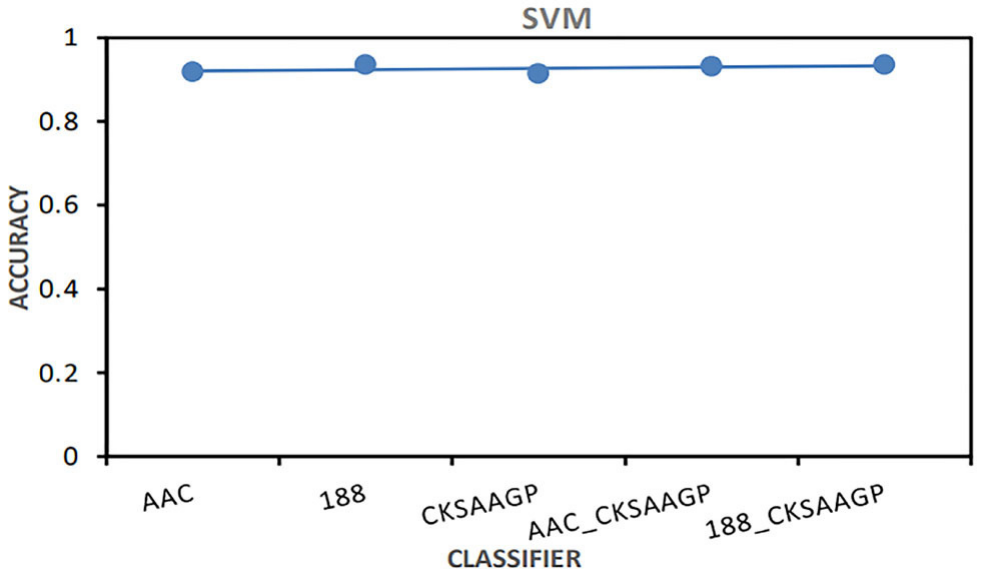
The AAC value obtained by the predictor constructed by SVM combined with five feature selection methods (AAC, 188-dimensions, CKSAAGP, AAC_CKSAAGP, and 188-CKSAAGP).

Among different feature extraction, Bagging predictor combined with 188-dimensions feature extraction to obtain the best prediction performance. NB, KNN and RF predictors combined with AAC feature extraction to obtain the best prediction performance. The prediction models built by SVM combined with 188-dimensions or 188_CKSAAGP feature extraction have obtained the best prediction results (Figure 4).

**Figure 5:**
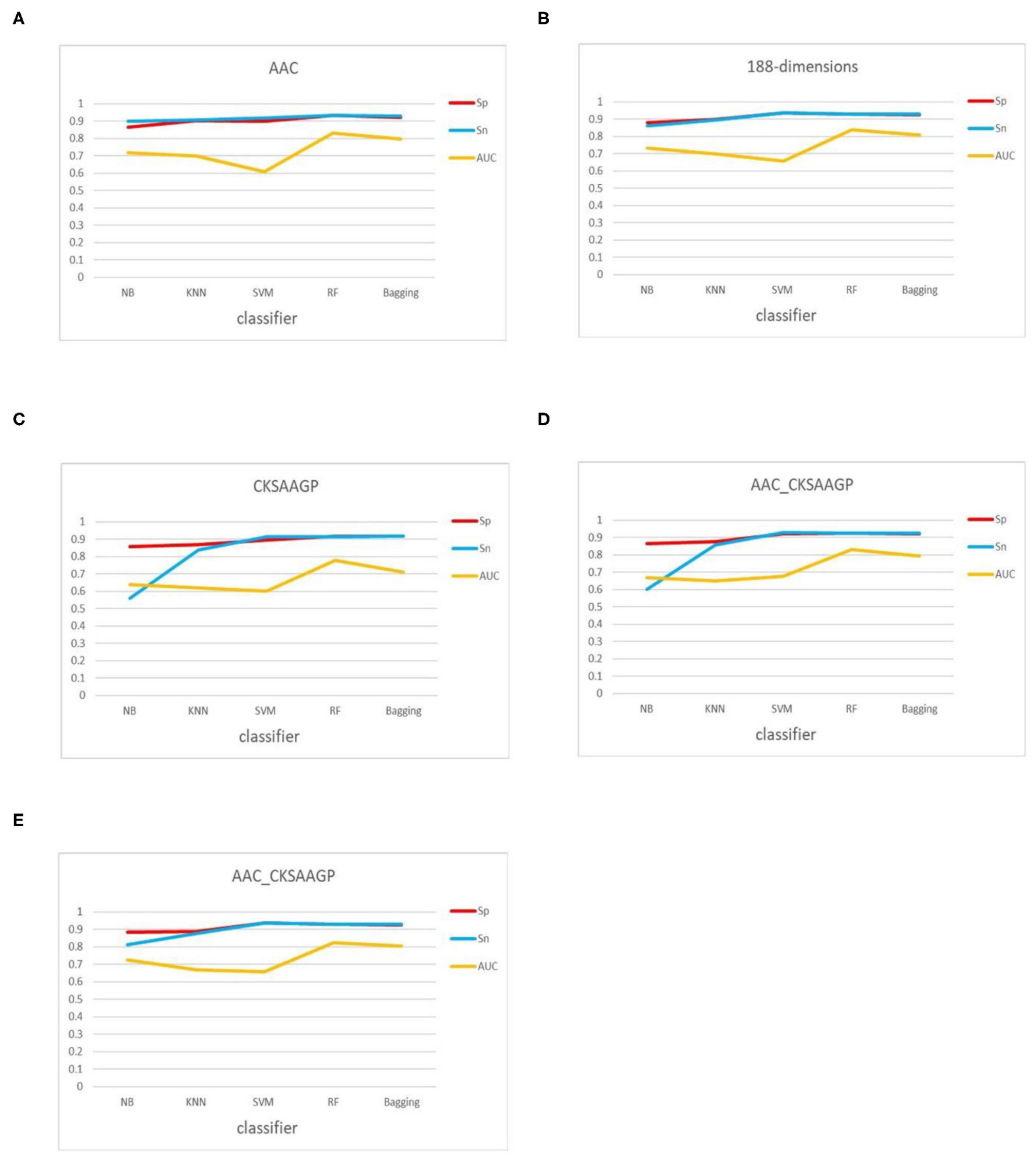
Sn, Sp, and AUC values obtained by 10-fold cross-validation through five feature extraction methods [**(A–E)** AAC, 188-dimensions, CKSAAGP, AAC_CKSAAGP, and 188-CKSAAGP] combined with five classifiers (NB, KNN, SVM, RF, Bagging).

In addition, the classification effect of the classifier constructed by 188-dimensions combined with different classification algorithms in terms of Sn, Sp, MCC, AUC is higher than other feature extraction methods, which proves that the prediction model of the former is better than other models (Figure 5). In the test datasets, SVM combined with 188-dimensions obtained a prediction accuracy of 92.77%, which proves that the prediction model is reliable. Therefore, in this study, we use 188-dimensions as the final feature extraction method.

## Conclusion

Rapid and accurate identification of tobacco mosaic virus is the key to successfully protecting tobacco from poison. Kumar and Prakash (2016) used direct antigen coating enzyme linked immunoassay (DAC-ELISA) technique to detect the TMV virus from pepper samples. However, this method is very complicated in sample preparation and detection processes, which is time-consuming and labor-intensive. Our goal was to distinguish between tobacco mosaic virus proteins and healthy tobacco leaf proteins in a large amount of data. The work in this paper provides an effective method to solve this problem.

In this experiment, first, we constructed a high-quality benchmark tobacco mosaic virus protein data set, which ensures the reliability of the classification tool. Secondly, we compared the performance of five feature extraction and five classifier constructs as predictors through 10-fold cross-validation, and then validated each model with the test datasets. The results show that SVM combined with 188-dimensions feature extraction method has the best prediction performance. It has obtained 93.58% accuracy on the train datasets and 92.77% accuracy on the test datasets, which proves that the prediction model has good robustness, so this paper chooses support vector machine as the prediction engine. We hope that these findings will help the development of identification of tobacco mosaic virus.

In future research, because feature selection technology has been successfully applied to some biological information experiments (Dong et al., 2015), feature selection on protein data can improve the prediction effect of the classifier. In addition, we will also try to use machine learning methods to solve analytical problems in genomics (Cheng et al., 2018c), epigenomics (Wang et al., 2017), and other proteomics fields.

## Data Availability Statement

Experimental data can be obtained from the corresponding author according to the reasonable request.

## Author Contributions

Y-MC collected the datasets. X-PZ processed the datasets. Y-MC and X-PZ designed the experiments. X-PZ did and analyzed the experiments' result. Y-MC contributed to the writing of this paper. All authors contributed to the article and approved the submitted version.

## Conflict of Interest

The authors declare that the research was conducted in the absence of any commercial or financial relationships that could be construed as a potential conflict of interest.
